# Big five model personality traits and job burnout: a systematic literature review

**DOI:** 10.1186/s40359-023-01056-y

**Published:** 2023-02-19

**Authors:** Giacomo Angelini

**Affiliations:** Department of Human Sciences, LUMSA University of Rome, 00193 Rome, Italy

**Keywords:** Personality, Personality traits, Burnout, Stress, Big five, PRISMA, Review

## Abstract

**Background:**

Job burnout negatively contributes to individual well-being, enhancing public health costs due to turnover, absenteeism, and reduced job performance. Personality traits mainly explain why workers differ in experiencing burnout under the same stressful work conditions. The current systematic review was conducted with the PRISMA method and focused on the five-factor model to explain workers' burnout risk.

**Methods:**

The databases used were Scopus, PubMed, ScienceDirect, and PsycINFO. Keywords used were: “Burnout,” “Job burnout,” “Work burnout,” “Personality,” and “Personality traits”.

**Results:**

The initial search identified 3320 papers, from which double and non-focused studies were excluded. From the 207 full texts reviewed, the studies included in this review were 83 papers. The findings show that higher levels of neuroticism (r from 0.10** to 0.642***; β from 0.16** to 0.587***) and lower agreeableness (r from − 0.12* to − 0.353***; β from − 0.08*** to − 0.523*), conscientiousness (r from -0.12* to -0.355***; β from − 0.09*** to − 0.300*), extraversion (r from − 0.034** to − 0.33***; β from − 0.06*** to − 0.31***), and openness (r from − 0.18*** to − 0.237**; β from − 0.092* to − 0.45*) are associated with higher levels of burnout.

**Conclusions:**

The present review highlighted the relationship between personality traits and job burnout. Results showed that personality traits were closely related to workers’ burnout risk. There is still much to explore and how future research on job burnout should account for the personality factors.

## Introduction

### Burnout: origin, evolution, and definition

Since the 1970s, when most research in occupational health psychology was focused on industrial workers, studies on burnout have seen a substantial increase. Initially considered a syndrome exclusively linked to helping professions [1, 2, 3, 4], burnout has been adopted by a broader range of human services professionals [5, 6]. Job burnout’s construct has undergone considerable conceptual and methodological attention in the last fifty years. Nowadays, job burnout is considered a multidimensional construct closely referred to as repeated exposure to work-related stress (e.g., [7]). According to the original theoretical framework, job burnout is defined chiefly as referring to feelings of exhaustion and emotional fatigue, cynicism, negative attitudes toward work, and reduced professional efficacy [6].

While the relationship between socio-demographic, organizational, and occupational factors and burnout syndrome have received significant attention, the relationship between burnout and individual factors, such as personality, is less explored (for a meta-analysis, see [8]).

Therefore, it is interesting to investigate whether there is sufficiently convincing evidence to indicate that personality factors play a role in predictors of job burnout. Investigating to what extent personality factors predict job burnout could include a measure of these factors in the selection processes of workers. At the same time, it could also allow preventive actions to support all those at risk of job burnout. This literature review involved a search for cohort studies published since 1993, which used self-report measures of personality traits and job burnout and investigated the relationships between these variables.

### Personality and job burnout

In the past, research on this issue has been chiefly haphazard and scattered ([9, 10] for a meta-analysis; [11]). Indeed, personality has often been evaluated in terms of positive or negative affectivity (respectively, e.g., [12, 13]), adopting the type A personality model (e.g., [14]), or the concept of psychological hardiness [15]. More recently, burnout research focused on the relationship between workers’ personalities measured by the Big Five personality model and their burnout syndrome [16, 17]. More specifically, neuroticism (e.g., [18, 19]) and extraversion personalities (e.g., [20]) were abundantly investigated in the scientific panorama (for review; [21]).

### Personality traits according to the five-factor model (FFM)

Since the twentieth century, scholars and researchers have increasingly dedicated themselves to studying this topic, given the importance assumed by personality in the psychological panorama. One of the most famous and relevant approaches to the study of character is the five-factor model (FFM) of personality traits (often referred to as the “Big Five”) proposed by McCrae & Costa [22, 23]. As a multidimensional set, personality traits include individuals’ emotions, cognition, and behavior patterns [23–26]. Furthermore, the FFM is the most robust and parsimonious model adopted to understand personality traits and behavior reciprocal relationships [27] due to two main reasons: its reliability across ages and cultures [28, 29] and its stability over the years [30]. According to several scholars, the FFM consists of five personality traits: agreeableness, conscientiousness, extraversion, neuroticism, and openness [23, 25, 26, 31]. Agreeableness refers to being cooperative, sympathetic, tolerant, and forgiving towards others, avoiding competition, conflict, pressuring, and using force [32]. Conscientiousness is reflected in being precise, organized, disciplined, abiding by principles and rules, and working hard to achieve success [33]. Extraversion is related to the quantity and intensity of individual social interaction characteristics. It is displayed through higher degrees of sociability, assertiveness, talkativeness, and self-confidence [32]. Neuroticism reflects people’s loss of emotional balance and impulse control. It is characterized by a prevalence of negative feelings and anxiety that are attempted to cope with through maladaptive coping strategies, such as delay or denial [29, 34]. Openness is reflected in intellectual curiosity, open-mindedness, untraditionality and creativity, the preference for independence, novelty, and differences [33, 35]. In the last thirty years, the Big Five model has been recognized as a primary representation of salient and non-pathological aspects of personality, the alteration of which contributes to the development of personality disorders [36–40], such as antisocial, borderline, and narcissistic personality disorders [41].

### Objectives

Although the role of the work environment as a predictor of burnout has been broadly documented (e.g., [5, 6, 11]), it cannot be neglected the effect that personality has on the development of this syndrome. Even reducing or eliminating stressors related to the work environment, some people may still experience high levels of burnout (e.g., [42]). For this reason, it is necessary to know the associations between personality traits and job burnout to identify the workers most prone to burnout and implement more risk-protection activities. Consequently, based on the literature presented above, this PRISMA review aimed to shed some light on the role that personality traits according to the Five Factors Model—Agreeableness, Conscientiousness, Extraversion, Neuroticism, and Openness—play in the development of job burnout.

## Methods

### Protocol and registration

**Fig. 1:**
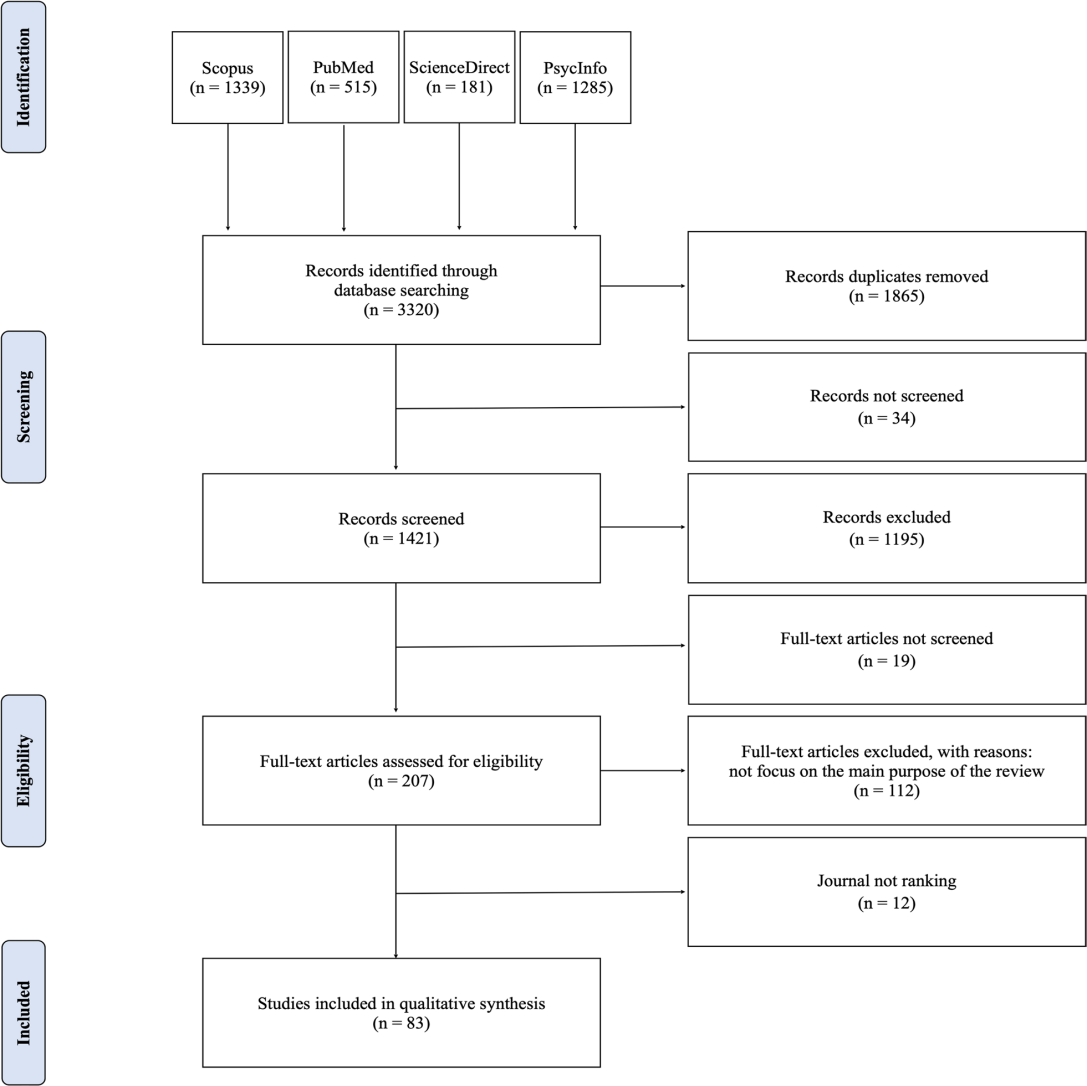
Diagram flow of information through the different phases of a systematic review

The systematic analysis of the relevant literature for this review followed procedures based on the Preferred Reporting Items for Systematic Reviews and Meta-Analyzes (PRISMA) process [43–45], a checklist of 27 items which together with a flow-chart (see Fig. 1) constitute the most rigorous guide to systematic reviews with or without meta-analysis. The systematic analysis of the relevant literature for this review followed procedures based on the Preferred Reporting Items for Systematic Reviews and Meta-Analyzes (PRISMA) process [43–45].

The PRISMA method intends to provide a checklist tool for creating systematic reviews of quality literature.

### Eligibility criteria

The study was conducted by extensively searching articles published before June 30th, 2021 (time of research), limited to papers in journals published in English. Review articles, meta-analyses, book chapters, and conference proceedings were excluded. Articles investigating the relationship between personality traits and job burnout in any field of employment, except athletic and ecclesiastical, were included.

### Information sources

The databases PsycINFO, PubMed, Scopus, and ScienceDirect, were used for the systematic search of relevant studies applying the following keywords:

* Burnout * AND * Personality *

* Burnout * AND * Personality traits *

* Job burnout * AND * Personality *

* Work burnout * AND * Personality *

* Job burnout * AND * Personality traits *

* Work burnout * AND * Personality traits *

The initial search identified 3320 papers. The details (title; author/s; year of publication; journal) of the documents identified for inclusion across all inquiries were placed in a separate excel document. After removing duplicates, reviewing titles, and reading abstracts (see Fig. 1), the papers were reduced to 207, of which full-text records were read. Studies selected in total for inclusion in this review were limited to the five dimensions of the Big Five Factor model [46] and were 83 papers.

## Results

### Study selection

As shown by the Prisma Diagram flow (Fig. 1), a total of 83 studies were identified for inclusion in the review. Via the initial search process have been identified total of 3320 studies (Scopus, n = 1339; PubMed, n = 515; ScienceDirect, n = 181; PsycInfo, n = 1285). After excluding duplicates, the remaining studies were 1455 of these 1421 records analyzed, and 1195 were discarded. After reviewing the abstracts, these papers did not meet the criteria. Of the remaining 226 full texts, the 207 papers available were examined in more detail, and it emerged that 112 studies did not meet the inclusion criteria as described. Furthermore, to ensure that only studies that had received peer review and met certain quality indicators were included, the SCImago Journal Rank (SJR) was inspected. SCImago considers the reputation and quality of a journal on citations, based on four quartiles used to classify journals from the highest (Q1) to the lowest (Q4). As suggested by Peters and colleagues [47], SCImago represents a widely accepted measure of the quality of journals and reduces the possibility of including in systematic reviews papers that do not meet certain quality indices. Based on this, 12 papers were excluded. Finally, 83 studies were included in the systematic review that met the inclusion criteria. Of the articles included in the review, more than half (60%) are published in journals indexed as Q1. The others were in Q2 (28%), Q3 (5%), and finally Q4 (7%).

### Study characteristics

#### Participants

The included studies have involved 36,627 participants. Based on the inclusion criteria, all reviewed studies included (1) adult samples (18 years or older), (2) workers from the general population rather than clinical samples, (3) regardless of the type of work, and for most studies (4) more female participants than male (female, 57.79%; male, 42.21%). Six studies did not include participants’ demographic information [48–53]. The above percentages refer to the available data (n = 33,299).

The sample consisted of about 26% Teachers or Professors, 22% Nurses, 11% Physicians with various specializations, 10% Policemen, 10% Health professionals, 8% Clerks, of which about 5% worked with IT. Furthermore, the sample was made up of almost 3% Drivers, and less than 2% ICT Manager and Firefighters. Finally, about 9% of the sample carried out different types of jobs.

#### Countries of collecting data

**Fig. 2:**
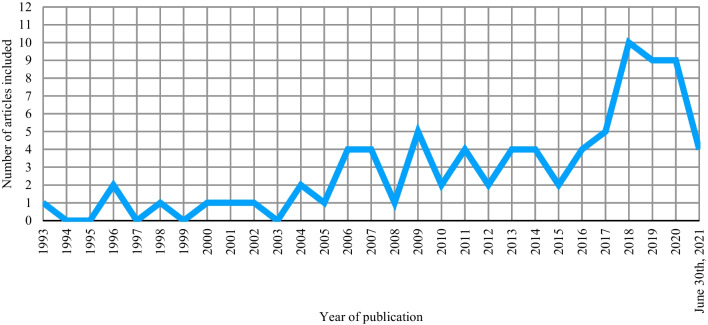
Research records achieving the inclusion criteria from 1993 to June 30th, 2021

The 83 articles included in this review have been published between 1993 and 2021 (see Fig. 2). In terms of geographic dispersion, more than half of the studies (n = 45; 54.21%) were conducted in Europe (France, Belgium, Bulgaria, Croatia, Germany, Greece, Italy, Netherland, Norway, Poland, Romania, Serbia, Spain, Sweden, Switzerland, and the UK). In contrast, the others were conducted either in America (n = 18; Canada, Jamaica, and the USA), Asia (n = 13; China, India, Iran, Israel, Jordan, and Singapore), Africa (n = 6; Nigeria, South Africa, and Turkey) and Oceania (n = 1; Australia).

A summary of information about the general characteristics and main methodological properties of all included 83 studies is reported in Table 1.

### Study selection

Concerning the key methodological features of studies, all studies reviewed involved empirical and quantitative research design. Most of the papers included (n = 73; 88%) in this review were cross-sectional and descriptive studies, except nine (11%) papers presenting longitudinal studies [50, 54–61]. Furthermore, one paper (1%; [62]) presented two different studies within it, one cross-sectional and the other longitudinal.

Most of the studies, 84% (n = 70), assessed job burnout via the Maslach Burnout Inventory, both in the original version (MBI; [3, 63]), and in the subsequent versions [64, 65], or its adaptation [66]. The other studies, 16% (n = 13), used tools other than MBI, but which share with it the theoretical approach to job burnout and the dimensions of (emotional) exhaustion, depersonalization or cynicism, and reduced personal or professional accomplishment (see Table 1). Five papers used the Shirom-Melamed Burnout Measure (SMBM; [67]), four the Oldenburg burnout inventory (OLBI; [68, 69]), one the Bergen Burnout Indicator (BBI; [70]), one the Brief Burnout Questionnaire (CBB; [71]), one the Burnout Measure [72] and one the Short Burnout Measure (SBM; [73]).

### Outcomes

According to the Big Five model, the outcome of the analyzed studies was the correlational and regressive between work burnout and personality traits. The data of the models in which the personality traits mediated or moderated the relationships with other variables, which were not the study’s object, were not considered in this review. Concerning personality, all included studies were compatible with the "Big Five" model [74, 75] and investigated traits of Agreeableness, Conscientiousness, Extraversion, Neuroticism, and Openness.

In detail, about 28% (n = 23) of the studies used the NEO Five-Factor Inventory (NEO-FFI; [33, 76–79]), 17% (n = 14) have used the Big Five Inventory (BFI; [31, 75, 80–83]), one of which is the 10-item version [84]. Yet, 10% (n = 8) used the Eysenck Personality Questionnaire (EPQ; [85, 86]), with one study with the revised version [87], and four studies with the revised and short version [88]. Furthermore, 7% (n = 6) involved the International Personality Item Pool (IPIP; [89, 90]), with two studies adopting the mini version [91], while another 7% (n = 6) involved the NEO-Personality Inventory (NEO-PI; [81]), with five studies adopting the revised version. About 5% (n = 4) has used the Ten-Item Personality Inventory (TIPI; [92]), 4% (n = 3) has used the Big Five mini markers scale [93], and 4% (n = 3) involved the Big Five Questionnaire (BFQ; [94]) Finally, about 2% (n = 2) has submitted the Five Factor Personality Inventory (FFPI; [95]), and 2% (n = 2) used the Mini Markers Inventory [93].

The remaining studies, about 14% (n = 12), used the following tools: the Basic Character Inventory (BCI; [96]), the Big Five factor markers [90], the Big Five measure-Short version [32, 97], the Big Five Plus Two questionnaire-Short version [98], the Brief Big five Personality Scale [92], the Basic Traits Inventory (BTI; [99]), the Comprehensive Personality and Affect Scales (COPAS; [100]), the Eysenck Personality Inventory (EPI; [101]), the Freiburg Personality Inventory (FPI; [102]), the M5-120 Questionnaire [103], the Minimal Redundant Scales (MRS-30; [104][104]), and the Personality Characteristics Inventory (PCI; [105, 106]).

All instruments included in the studies were in line with the “Big Five” domains [26], such as e.g., the NEO-FFI and the NEO-PI, widely used measures of the Big Five [81], the dimensions of the TIPI and the IPIP [89, 92], or the factors of the EPQ and the EPI, compatible with the Big Five model [107, 108].

### Risk of bias in individual studies

Study design, sampling, and measurement bias were assessed regarding the evaluation risk of bias in each study. Table 2 summarizes the limits reported in each study. Where not registered, no limitations related to the study were referred by the authors of the original studies.

### Study design bias

Although most of the studies (89%) have a cross-sectional design, this review reported in the table (see Table 2) this bias only on the studies that highlighted this as a weakness (50%). Cross-sectional methods are cheap to conduct, agile for both the researcher and the participant, and can give answers to many research questions [109]. At the same time, however, since it is a one-time measurement, it does not allow us to test dynamic and progressive effects to conclude the causal relationships among variables.

Three longitudinal studies reported a shortness [56, 58] or longness [55] time-lag between the first and successive administrations. The time length between the study’s waves is an essential issue in longitudinal research methodology. The time interval between the first and following measurements should correspond with the underlying causal lag (e.g., [110]). If the time lag is too short, probably the antecedent variable does not affect the outcome variable. If, on the contrary, the time lag is too long, the effect of the antecedent variable may already have disappeared. In both cases, the possibility of detecting the impact of the antecedent variable on the outcome variable may decrease.

**Table 1:** The included studies

References	Study design	Study sample	Country	Job	Female	M_age_	SD_age_	Burnout measures	Personality measures	Years
Manlove [111]	CSS	188	USA	Child care workers	98.94%	34	DNA	MBI	EPI	1993
Deary et al. [48]	CSS	375	UK	Medical staff	DNA	DNA	DNA	MBI	NEO-PI-R	1996
Deary et al. [112]	CSS	188	UK	Physicians/surgeons	9.4%	47.0	1.2	MBI	NEO-FFI	1996
Mills et al. [60]	LS	225	USA	School psychologists	73.4%	40.3	9.3	MBI	NEO-FFI	1998
Zellars et al. [113]	CSS	188	USA	Hospital nurses	89.9%	40	8.0	MBI	NEO-FFI	2000
Zellars et al. [53]	CSS	296	USA	Hospital nurses for acute care	DNA	42.2	9.5	MBI	NEO-FFI	2001
De Vries et al. [114]	CSS	765	Netherland	Workers	46.5%	40.3	9.7	MBI	FFPI	2002
McManus et al. [50]	LS	1668	UK	Doctors	DNA	30.4	1.86	MBI	BFQ	2004
Zellars et al. [52]	CSS	290	USA	Hospital nurses for acute care	DNA	42.19	9.45	MBI	NEO-FFI	2004
Cano-García et al. [115]	CSS	99	Spain	Teachers	74%	42.5	8.5	MBI	NEO-PI-R	2005
Burke et al. [116]	CSS	496	Norway	Nursing home employees	89.1%	DNA	DNA	BBI	BFI	2006
Goddard et al. [57]	LS	79	Australia	Beginning teachers	84%	26	7.46	MBI-ES	EPQ-RS	2006
Langelaan et al. [117]	CSS	572	Netherland	Employees	17%	42	8.0	MBI-GS	NEO-FFI	2006
Mostert et al. [118]	CSS	1749	South Africa	Policeman	18.1%	34.53	6.23	MBI-GS	PCI	2006
Bahner et al. [119]	CSS	115	USA	BIP workers	56%	43	11.77	MBI	COPAS	2007
Ghorpade et al. [120]	CSS	265	USA	Professors	46.39%	50	10.24	MBI-ES	Mini-Markers Inventory	2007
Kim et al. [121]	CSS	191	USA	Hotels employees	62.3%	36	DNA	MBI-GS	IPIP	2007
Teven [122]	CSS	48	USA	Professors	43.75%	51.15	9.86	MBI	Big Five measure-SF	2007
Leon et al. [123]	CSS	203	USA	Children’s RTCs	63.50%	31.64	9.41	MBI	BFI	2008
Chung et al. [124]	CSS	103	UK	Residential community staff	70%	37.51	10.97	MBI	NEO-FFI	2009
De Hoogh et al. [125]	CSS	91 190	Netherlands	Clients of a human resource Employees	43% 59%	42 36	DNA DNA	MBI-GS	IPIP	2009
Gandoy-Crego et al. [126]	CSS	42	Spain	Geriatric nurses	84%	31	5.7	MBI	BFQ	2009
Kim et al. [127]	CSS	187	USA	Subway employees	67%	22	DNA	MBI-GS	IPIP	2009
Taormina et al., [128]	CSS	172	China	Casino workers	40.12%	27.87	5.17	MBI	NEO-PI-R	2009
Barford et al. [129]	CSS	94	Canada	Child and youth care workers	69.1%	32.82	9.75	MBI-GS	NEO-FFI	2010
Perry et al. [51]	CSS	252 392	USA	Customer service providers Manual laborers for repair and construction services	83% DNA	34.7 DNA	9.83 DNA	Exhaustion scale MBI-GS	Personal Characteristics Inventory Big Five factor markers	2010
Ghorpade et al. [130]	CSS	263	USA	Professors	46.4%	50.14	10.24	MBI	Mini-Markers Inventory	2011
Hudek-Knežević et al. [59]	LS	118	Croatia	Hospital nurses	100%	36.47	7.02	MBI	BFI	2011
Salami [131]	CSS	340	Nigeria	Teachers	29.42%	36.70	4.50	MBI-GS	NEO-FFI	2011
Sterud et al. [61]	LS	298	Norway	Ambulance workers	16.9%	38.2	8.9	MBI-HSS	BCI	2011
Armon et al. [54]	LS	1105	Israel	Health workers	37%	DNA	DNA	SMBM	Big-Five mini markers scale	2012
Zimmerman et al. [132]	CSS	587	USA	Employees	11%	49	DNA	MBI	Big-Five mini markers scale	2012
De la Fuente Solana et al. [133]	CSS	747	Spain	Policeman	11.8%	35.7	8.33	MBI	NEO-FFI	2013
Garbarino et al. [134]	CSS	289	Italy	Policeman	0%	35.4	7.5	MBI	BFQ	2013
Hurt et al. [135]	CSS	113	USA	ABA therapists	95.6%	DNA	DNA	MBI-GS	M5-120	2013
Lin et al. [136]	CSS	228	China	Employees	19.79%	27.9	3.9	MBI	EPQ	2013
Gan et al. [56]	LS	160	China	IT employees	36.2%	27.78	3.91	MBI-GS	NEO-FFI-SF	2014
Reinke et al. [137]	CSS	201	UK	Workers	51.74%	34.78	9.49	OLBI	TIPI	2014
Taycan et al. [138]	CSS	139	Turkey	Physicians	33.1%	31.05	4.84	MBI-HSS	EPQ-RS	2014
Yilmaz, [139]	CSS	303	Turkey	Teachers	53.5%	DNA	DNA	MBI	Mini-IPIP	2014
Cañadas-De la Fuente et al. [140]	CSS	676	Spain	Nurses	66%	44.58	8.18	MBI	NEO-FFI	2015
Srivastava et al. [141]	CSS	152	Europe and Asia	Senior organizational managers who regularly use ICT	23.7%	37,96	6,73	MBI	TIPI	2015
Ang et al. [142]	CSS	1826	Singapore	Nurses	61.5%	DNA	DNA	MBI	NEO-FFI	2016
Iorga et al. [143]	CSS	37	Romania	Forensic physicians	54.05%	39.13	11.78	MBI	BFI	2016
Vaulerin et al. [144]	CSS	220	France	Firefighters	0%	36.23	6.94	SMBM	BFI	2016
Zhou et al. [145]	CSS	1129	China	Physicians	58.17%	38.04	7.74	MBI	EPQ-RS	2016
De la Fuente-Solana et al. [146]	CSS	101	Spain	Oncology nurses	69.3%	DNA	DNA	MBI	NEO-FFI	2017
Geuens et al. [147]	CSS	587	Belgium	Nurses	82%	40	10.8	MBI	NEO-FFI	2017
Iorga et al. [148]	CSS	116	Romania	Obstetrics and gynecology physicians	69.83%	DNA	DNA	MBI	BFI	2017
Lovell et al. [149]	CSS	120	UK	Prison officers	40.7%	41.72	10.73	MBI-HSS	NEO-PI	2017
Ntantana et al. [150]	CSS	149 320	Greece	Physicians ICU nurses	33.4% 19.2%	DNA DNA	DNA DNA	MBI	EPQ	2017
Al Shbail et al. [151]	CSS	187	Jordan	Internal auditors	7.5%	DNA	DNA	BM	NEO-PI-R	2018
Bergmüller et al. [152]	CSS	97	Germany	Ambulance doctors	41.24%	37.0	12.21	MBI-GS	FPI	2018
Bianchi et al. [153]	CSS	257	Switzerland	Teachers	76%	44.84	10.46	SMBM	NEO-FFI	2018
Bianchi, [19]	CSS	1759	France	Teachers	77%	40.81	9.63	SMBM	NEO-FFI	2018
Harizanova et al. [49]	CSS	307	Bulgaria	Correctional officers	DNA	DNA	DNA	MBI	EPQ	2018
Hildenbrand et al. [58]	LS	148	Germany	Employees of a manufacturing company	22%	DNA	DNA	OLBI	MRS-30	2018
Iorga et al. [154]	CSS	78	Romania	Hospital pharmacists	89.7%	45.57	10.12	MBI	BFI	2018
Tang et al. [155]	CSS	862	China	Clinical health professionals	80.4%	DNA	DNA	MBI-HSS	Brief Big five Personality Scale	2018
Tatalović Vorkapić et al. [156]	CSS	203	Croatia	Educators	100%	38.73	10.69	Scale of professional burnout of educators	BFI	2018
Yao et al. [157]	CSS	860	China	Nurses	94.42%	DNA	DNA	MBI-GS	EPQ-RS	2018
Zaninotto et al. [158]	CSS	215	Italy	Mental health professionals	59.1%	46.98	8.09	MBI	TIPI	2018
Bahadori et al. [159]	CSS	308	Iran	Technicians of emergency medical personnel	0%	30	5.43	MBI	NEO-FFI	2019
Brown et al. [160]	CSS	77	Canada and Jamaica	Primary care physicians	79%	DNA	DNA	MBI-HSS	BFI	2019
Castillo-Gualda et al. [62]	CSS LS	237 59	Spain	Teachers	65.4% 66.10%	44.32 41.12	10.54 9.91	MBI-ES	BFI	2019
De la Fuente-Solana et al. [161]	CSS	96	Spain	Oncology nurses	68.8%	45.5	8.02	MBI	NEO-FFI	2019
De Looff et al. [55]	LS	110	Netherland	Nurses for forensic psychiatric hospitals	59%	35.5	10.0	MBI	NEO-FFI	2019
Farfán et al. [162]	CSS	237	Spain	Workers of State Security Forces and Corps	24.05%	37.72	DNA	MBI-GS	NEO-PI-R	2019
Khedhaouria et al. [163]	CSS	161	France	Senior managers who regularly use ICT	49.61%	39	DNA	SMBM	TIPI	2019
Pérez-Fuentes et al. [17]	CSS	1236	Spain	Nurses	85.5%	31.50	6.18	CBB	BFI-10	2019
Ye et al. [164]	CSS	622	China	HSR drivers	0%	37.2 DNA	2.31 DNA	MBI-GS	BFI	2019
Banasiewicz et al. [165]	CSS	181	Poland	Midwives participating and non-participating in pregnancy terminations	100%	40.79	8.55	OLBI	EPQ-R	2020
Bhowmick et al. [166]	CSS	152	India	Policeman	2%	43.4	9.34	MBI	Big-Five mini markers scale	2020
De Vine et al. [167]	CSS	127	South Africa	Workers	64%	33.21	12.17	MBI-GS	BTI	2020
Dionigi, [168]	CSS	160	Italy	Clown doctors	72,5%	38.63	11.42	MBI	BFI	2020
Farfán et al. [169]	CSS	971	Spain	Workers	56.95%	37.58	DNA	MBI	Mini-IPIP	2020
Liu et al. [170]	CSS	451	China	Employee-supervisor dyads	19.82%	33.16	9.49	MBI-GS	IPIP	2020
Mahoney et al. [171]	CSS	246	USA	Nurse anesthetists	60%	48.03	11.34	OLBI	TIPI	2020
Malka et al. [172]	CSS	311	Israel	Social workers	90%	42.8	8.9	SBM	BFI	2020
Tasic et al. [173]	CSS	302	Serbia	Nursery teachers	100%	38	9.2	MBI-GS	Big Five Plus Two questionnaire-SF	2020
Bianchi et al. [174]	CSS	4394 611 514	France Spain Swiss	Teachers	86% 70% 68%	44.78 45.98 44.95	9.35 9.39 10.54	MBI-ES	NEO-FFI	2021
De la Fuente-Solana, Pradas-Hernández, González-Fernández, et al. [175]	CSS	94	Spain	Paediatric nurses	78.7%	43.89	10.50	MBI-HSS	NEO-FFI	2021
De la Fuente‐Solana, Suleiman‐Martos, Velando‐Soriano, et al.,[176]	CSS	150	Spain	Midwives and nurses	78.7%	44.85	11.563	MBI	NEO-FFI	2021

ABA Applied Behaviour Analysis; BBI Bergen Burnout Indicator; BCI Basic Character Inventory; BFI, Big Five Inventory; BFQ, Big Five Questionnaire; BIP, Batterer Intervention Program; BM, Burnout Measure; BTI, Basic Traits Inventory; CBB, Brief Burnout Questionnaire; COPAS, Comprehensive Personality and Affect Scales; CSS, Cross-sectional study; DNA, Data Not Available; EPI, Eysenck Personality Inventory; EPQ-R, Eysenck Personality Questionnaire Revised; EPQ-RS, Eysenck Personality Questionnaire Revised Short Scale; EPQ, Eysenck Personality Questionnaire; FFPI, Five-Factor Personality Inventory; FPI, Freiburg Personality Inventory; ICT, Information and Communication Technologies; ICU, Intensive Care Unit; IPIP, International Personality Item Pool; IT, information technology; LS, Longitudinal study; M, Mean; MBI-ES, Maslach Burnout Inventory-Educators Survey; MBI-GS, Maslach Burnout Inventory-General Survey; MBI-HSS, Maslach Burnout Inventory-Human Services Survey; MBI, Maslach Burnout Inventory; MRS, Minimal Redundant Scales; NEO-FFI-PI-R, Neuroticism Extraversion Openness Five Factor Inventory Personality Inventory-Revised; NEO-FFI-PI, Neuroticism Extraversion Openness Five Factor Inventory Personality Inventory; NEO-FFI, Neuroticism Extraversion Openness Five-Factor Inventory; NEO-PI, Neuroticism Extraversion Openness Personality Inventory; NEO-PI-R, Neuroticism Extraversion Openness Personality Inventory Revised; OLBI, Oldenburg Burnout Inventory; PCI, Personality Characteristics Inventory; RTC, Residential Treatment Center; SD; Standard Deviation; SF, Short Form; SMBM, Shirom-Melamed Burnout Measure; TIPI, Ten-Item Personality Inventory

**Table 2:** Main study limitations and risk of biases in the 83 studies reviewed

References	CSS	PM	SSS	NPS	LRR	SIM	PMFS	PRS	TLSL	CGB	SSOWS	SVL	Other limitations	Years
Manlove [111]	X	X												1993
Deary et al. [48]	X												The use of zero-order statistics for the evaluation of multifactorial constructs, such as psychological issues, has been recognized as being inappropriate	1996
Deary et al. [112]														1996
Mills et al. [60]		X		X							X		The exclusion of the personality measure at Time 2 did not allow for a complete analysis of the effects of personality differences on burnout dimensions	1998
Zellars et al. [113]	X	X												2000
Zellars et al. [53]	X	X			X									2001
De Vries et al. [114]														2002
McManus et al. [50]														2004
Zellars et al. [52]	X	X			X							X		2004
Cano-García et al. [115]														2005
Burke et al. [116]	X	X						X						2006
Goddard et al. [57]			X	X									Participants may have different characteristics from those of those who did not participate No comparison with a control group	2006
Langelaan et al. [117]	X							X						2006
Mostert et al. [118]	X	X												2006
Bahner et al. [119]	X			X									The focus of the study was on the male batterer intervention program in the context of heterosexual relationships, not that of same-sex relationships It is not known whether the results may reflect the different nature and structure of the working environments of the batterer intervention programs	2007
Ghorpade et al. [120]														2007
Kim et al. [121]														2007
Teven [122]		X	X								X		The data comes from a sample with only the level of education university Respondents may have tried fatigue because many of the measures overlap conceptually	2007
Leon et al. [123]		X				X		X					There are missing data and differences between the samples for age and education No specific job characteristics to RTCs were explored that might be associated with the client presentations measured It is possible that this study's moderation findings will not generalize to other samples of front line staff	2008
Chung et al. [124]													Challenging behavior has been measured in terms of the level of challenging behavior of the clients with whom they worked most intensively, and this does not accurately or reliably reflect exposure to challenging behavior No data on staff views of their job, on assaults or witnessed violence by staff, or on the kind of recent significant life events of the staff	2009
De Hoogh et al. [125]	X		X										In Sample 1 ratings of independent and dependent variables were provided by the same subordinates In Sample 2 the selection of raters may have selected employees who share attitudes and opinions	2009
Gandoy-Crego et al. [126]			X											2009
Kim et al. [127]		X	X								X		Focus limited to individual differences	2009
Taormina et al. [128]			X					X				X		2009
Barford et al. [129]	X	X	X					X					Different methods of data collection were used	2010
Perry et al. [51]	X	X										X	The distinct contexts across the two samples and lower variance across variables in Sample 1 may have affected the results	2010
Ghorpade et al. [130]														2011
Hudek-Knežević et al. [59]		X	X										Burnout at Time 1 was not measured Sample attrition due to drop-out at Time 2 Significant differences (age, agreeableness, and continuance commitment) were found between the sample included and the one that in both Times and the one that dropped out	2011
Salami, [131]	X	X												2011
Sterud et al. [61]														2011
Armon et al. [54]													The findings could be biased because of the "healthy worker effect" Longitudinal design was based on only two waves of measurement The personality scale only return a total score and do not evaluate the influence of the personality variables on each of the burnout factors The possibility that the factors of the personality may interact in affecting burnout levels has not been tested	2012
Zimmerman et al. [132]		X											The sample was composed exclusively of high-level professionals Only neuroticism and extraversion and not all personality traits were evaluated	2012
De la Fuente Solana et al. [133]	X													2013
Garbarino et al. [134]	X	X	X											2013
Hurt et al. [135]														2013
Lin et al. [136]	X	X											The sample was relatively young Questionnaires used are not popular therefore the comparison with other studies (also of other nationalities) may be limited The employees who did not respond to our questionnaire might have been those who were busier	2013
Gan et al. [56]			X	X					X				Could not examine the reciprocal relationship (burnout/engagement to demands/resources) using panel data	2014
Reinke et al. [137]	X	X										X		2014
Taycan et al. [138]	X												Lack of an urban physician sample for comparison	2014
Yilmaz, [139]														2014
Cañadas-De la Fuente et al. [140]	X		X	X										2015
Ang et al. [142]	X	X			X									2015
Iorga et al. [143]	X										X			2016
Vaulerin et al. [144]			X											2016
Zhou et al. [145]	X	X											Some scales have been adapted to the sample under study and their construct validity and temporal stability have not been confirmed The questionnaire was completed on workdays and may have caused additional stress for the firefighters	2016
De la Fuente-Solana et al. [146]	X	X									X			2016
Geuens et al. [147]	X		X		X									2017
Iorga et al. [148]	X	X		X	X								The study was conducted from November 2012 to July 2013 and could lead to different results than a recent one The focus was only on individual factors, leaving out job-related and organizational factors	2017
Lovell et al. [149]	X		X									X	The access to medical facilities and the small distance from patients who live in urban areas may have diminished the physicians' professional exhaustion	2017
Ntantana et al. [150]	X			X									Methods based on measuring physiological parameters are not objective	2017
Al Shbail et al. [151]	X				X	X							Causal factors have not been investigated	2017
Bergmüller et al. [152]		X										X	Only companies that were part of the Amman Stock Exchange (ASE) were included in the study and not the others All types of shareholding companies were included in the study, regardless of sub-sectors	2018
Bianchi et al. [153]														2018
Bianchi, [19]	X	X									X		It was used only one measure of burnout The role of organizational and work-contextualized factors may have been overemphasized in burnout research, to the detriment of the role of generic dispositional factors Burnout's discriminant validity is unsatisfactory	2018
Harizanova et al. [49]	X			X									An exhaustion-centered measure of burnout was used Only neuroticism and extraversion and not all personality traits were evaluated	2018
Hildenbrand et al. [58]	X													2018
Iorga et al. [154]		X							X		X		The correlation between thriving and burnout at Time 1 and Time 2 was rather high Despite large groups of employees sharing the same supervisor, it was not possible to collect information on group composition and therefore take into account the variance due to team membership	2018
Tang et al. [155]							X			X				2018
Tatalović Vorkapić et al. [156]	X										X		The psychological distress of the participants was not measured and also the fitting degrees of both logistic regression modes are low, suggesting that there are other unknown risk factors	2018
Yao et al. [157]			X					X						2018
Zaninotto et al. [158]													The participants of the sample, coming from the municipal hospitals, had heavy patient loads and therefore more exposed to burnout	2018
Bahadori et al. [159]	X	X											A large number of correlations increases the risk of type 1 errors	2018
Brown et al. [160]	X	X										X		2019
Castillo-Gualda et al. [62]	X				X		X							2019
De la Fuente-Solana et al. [161]	X^1^	X	X^2^	X^2^								X		2019
De Looff et al. [55]	X		X	X										2019
Farfán et al. [162]			X						X				Methods based on measuring physiological parameters are not objective	2019
Khedhaouria et al. [163]		X	X								X	X		2019
Pérez-Fuentes et al. [17]	X							X					Organizational factors that reduce stress, the effect of technostress, and personality traits on job burnout among employees, nor cases that could have specific antecedent conditions predisposing job burnout were not evaluated	2019
Ye et al. [164]													Some information is lost due to some scales, which are too short to adequately evaluate the personality construct, or they only return a total burnout score and do not evaluate the influence of the personality variables on each of the burnout factors	2019
Banasiewicz et al. [165]	X	X											The Baron and Kenny method to examine the mechanism of the mediator is too simple	2019
Bhowmick et al. [166]			X										The questionnaire contained closed-ended type questions The questionnaire required a long duration of completion Pregnancy terminations are performed only at several facilities in Poland	2020
De Vine et al. [167]		X		X	X	X	X	X				X		2020
Dionigi, [168]			X				X							2020
Farfán et al. [169]	X		X							X			The predictors evaluated accounted for only a part of the burnout dimensions	2020
Liu et al. [170]	X			X							X		Sample with high concentration of people with a high education level	2020
Mahoney et al. [171]		X								X			The study focuses on negative workplace gossip and does not include positive workplace gossip The scale of negative workplace gossip is based on frequency, but the perception of negative workplace gossip may have different influences The mean score for negative workplace gossip is relatively lower than those of other studies with Chinese samples	2020
Malka et al. [172]														2020
Tasic et al. [173]		X											The issue of work supervisors who wanted to leave the job was not evaluated Being a quantitative study, it lacks the interpretive perspective of the participants It is not clear to what degree participants related to their role as fieldwork supervisor compared to their experiences workers in general	2020
Bianchi et al. [174]														2020
Fuente-Solana et al. [175]	X	X		X		X	X				X	X	French sample was considerably larger than Spanish and Swiss samples	2021
Fuente–Solana et al. [176]			X	X								X	Methods based on measuring physiological parameters are not objective There was the impression that a sample with a certain level of burnout was more prone to participate	2021
Manlove [111]	X		X	X								X	Methods based on measuring physiological parameters are not objective	2021

CSS, Cross-Sectional; PM, Perceptual Measures; SSS, Small Sample Size; NPS, Non-Probabilistic Sampling; LRR, Low Response Rate; SIM, Single-Item Measures; PMFS, Predominantly Male/Female Sample; PRS, Poor reliability scale; TLSL, Time Lag too Short/Long; CGB, Cultural/Geographical Bias; SSOWS, Sample of a Single Organization/Work/State; SVL, Study Variables were Limited

Furthermore, it is possible that in the period between the first and subsequent measurements, several events may occur affecting the outcome. Finally, the same participant in the sample could change the condition under study (to know more, [177]). Especially in work-related studies, employees may be subject to changes in context, needs, and working hours [178]. Despite this, longitudinal designs offer substantial advantages over cross-sectional methods in examining the causal links between the variables [177].

### Sampling bias

About 29% of the studies (n = 24) reported the small samples as limitation. Among these, one study that had two different samples reported a small sample only in second one [62], while another study, in investigating differences, highlighted that certain groups have a relatively small sample size and reported this as a limitation [140]. Additionally, about 10% of the studies reported having received an inadequate response rate. About 18% of the studies reported a non-probabilistic sampling as a limitation, and 6% of studies examined reported having a gender-biased sample (male/female). Other studies (13%) reported collecting data in a single organization, country, or an imbalance among workers’ categories. Finally, three studies [154, 168, 170] reported a cultural or geographical bias. To sum up, studies’ limitations regarding the sample characteristics may significantly impact scores’ reliability [179, 180]. Specifically, this research’s limits prevent to generalize the findings. 

### Measurement and response bias

Since inclusion evaluated burnout and personality traits through self-reports that respected the previously illustrated models, all the studies examined used self-report measures. Again, only 40% report this as a limitation. Using perceptual measures, one could be subject to the Common Method Bias (CMB; [181]). The CMB occurs when the estimated relationships among variables are biased due to a unique-measure method [182]. This bias may be due to several factors, including response trends due to social desirability, similar responses of respondents due to proximity and wording of items, and similarity in the conditions of time, medium, and place of measurements [183–185]. These variations in responses are artificially attributed to the instrument rather than to the basic predispositions of the participants [181, 186, 187]. Suppose the systematic method variance is not contained. In that case, it can result in an incorrect evaluation of the scale's reliability and convergent validity, inflating the reliability estimates of correlations [188] and distorting the estimates of the effects of the predictors in the regressions [184].

Furthermore, about 5% of studies reported using single-item measures. Personality characteristics were often measured through self-reports with single items and assessed through a Likert scale [189]. This type of assessment is susceptible to social desirability (SDR; [184, 185]), i.e., the tendency to respond coherently with what others perceive as desirable [190]. Furthermore, this type of assessment is also susceptible to acquiescent responding (ACQ; [191]), i.e., the tendency to prefer positive scores on the Likert scale, regardless of the meaning of the item [192]. Response-style-induced errors can influence reliability estimates (e.g., [193, 194]) and overestimate or underestimate the relationships between the variables examined [195]. Despite these response biases, widely documented in the literature [184–186, 196–198], it appears that this bias is overstated in psychological research [185]. Indeed, self-reports would seem to be the most valid measurement method for evaluating personality factors because the same participant is the most suitable person to report their personality and level of burnout [42]. Other studies (10%) reported using a poor reliability scale: employing imprecise psychometric procedures in a study is likely to distort the outcome, therefore not allowing to make inferences about an individual and creating a response bias [199]. Finally, about 16% of the studies examined reported that the study did not review all the variables relating to the constructs investigated. Table 2 also identifies some specific limitations of the studies examined, such as, e.g., the comparison between non-numerically equivalent samples [174], the long compilation time required [165], and the lack of a control group [57, 138]. Furthermore, some studies have used tools that evaluate only a total score of burnout [17] or personality [54] Finally, other studies have focused only on individual factors, leaving out job-related and organizational factors [147].

This systematic review was conducted to identify, categorize, and evaluate the studies investigating the relationship between job burnout and personality traits addressed to date. Specifically, the interest of this review was to explore the role of personality traits as individual factors related to job burnout. To do this, only studies that analyzed the direct relationship between personality traits and job burnout were included, leaving out all those studies that investigated additional variables that could in any way mediate or moderate this relationship.

## Results of the studies included

Table 3 summarizes the results, the correlation and regression indices, and the power of significance of the studies included in this review.

The results of the included studies based on the five personality traits and the association with a dimension of job burnout are discussed below. The correlations between the personality trait and the size of the job burnout report first, while subsequently those of the regressions, presenting the cross-sectional studies first, which are most of them, and then also the longitudinal ones.

## Discussion

As seen previously, job burnout is a multidimensional construct that consists of the individual response to stressors at work [3, 9]. The literature has long investigated the association between organizational and occupational factors and burnout. However, a recent meta-analysis shows that there is a bidirectional relationship between occupational stressors and burnout [200]. Because the research on individual factors has been less systematic, partial, and contradictory [113], this review aimed to synthesize research evidence about the role that FFM personality traits play in the development of job burnout. To do this, 83 independent studies that used different tools to assess both job burnout and personality traits while maintaining the same reference theory were identified. The most investigated personality traits were, in order, neuroticism, extraversion, agreeableness, conscientiousness, and openness to experience.

The present review extracted data from the reviewed studies, including (1) main characteristics of participants (including job type), (2) data collected country, (3) personality traits related to job burnout, (4) risk of bias in individual studies, and (5) methodological features of studies. As for the participants, all reviewed studies included (1) adult samples, (2) workers from the general population rather than clinical samples, (3) regardless of the type of work, and for most studies (4) more female participants than male. Based on these observations, future studies examining personality traits and work burnout should employ other samples (e.g., clinical samples) to enhance external validity.

This systematic review focused exclusively on personality traits and the relationship between them and job burnout. Results of the included studies confirmed a relationship between job burnout and the five distinct personality traits of the Big Five model [46] and that some of these were risk factors for job burnout (although not always in the same direction). A descriptive picture of the relationship between the five personality traits and job burnout will be discussed.

### Agreeableness

A negative association between Agreeableness and job burnout was reported (range, r from − 0.12* to − 0.353***; β from − 0.08*** to − 0.523*). Longitudinal studies also suggest a role of Agreeableness as a protective factor of dimensions of Emotional Exhaustion, Depersonalization, and reduced Professional Accomplishment (EE; β, − 0.83*; β, − 0.48*; D; β, − 0.31*; PA; β, − 0.22*; rPA; β, − 0.28**). As seen previously, the Agreeableness trait has been described as a sense of cooperation, tolerance, and avoidance of conflict on problematic issues [32]. Agreeable individuals are warm, supportive, and good-natured [201, 202], protecting them from feelings of frustration and emotional exhaustion [113]. Indeed, their tendency towards a positive understanding of others, coupled with interpersonal relationships based on feelings of affection and warmth [201], could protect them from developing job burnout and greater depersonalization [8, 203]. Although most of the studies found a negative relationship between Agreeableness and job burnout, in some studies Agreeableness was positively correlated with Emotional exhaustion [159], and reduced Professional Accomplishment [50, 62].

### Conscientiousness

A negative association between Conscientiousness and job burnout was reported (range, r from − 0.12* to − 0.355***; β from − 0.09*** to − 0.300*). Longitudinal studies also suggest the role of Conscientiousness as a protective factor against Burnout (B; β, -0.21*). As seen previously, the Conscientiousness trait is reflected in precise, organized, and disciplined individuals who respect the rules and work hard to achieve success [33]. Their perseverance in work and success orientation would protect these people from developing emotional exhaustion [76, 204] and poor personal accomplishment, as they are unlikely to perceive themselves as unproductive. Although most studies found a negative relationship between Conscientiousness and job burnout dimensions, some studies pointed out an unexpected inverse correlation between Conscientiousness and reduced Professional Accomplishment [60, 62, 143, 159, 166]. Furthermore, Conscientiousness was positively associated with Emotional exhaustion and Depersonalization [131]. This result would be due to the greater commitment and effort employed in their work, which would have greater levels of exhaustion and depersonalization [131]. Finally, another longitudinal study [56] attributes Conscientiousness as a negative predictor role for the dimensions of Personal/Professional Accomplishment. However, the authors do not provide reasons for this discordant result from the literature.

**Table 3:** Results of the variables reviewed in the 83 studies reviewed

	Correlation					Regression					
References	N	E	A	C	O	N	**E**	A	C	O	Years
Manlove [111]	+ EE (r, 0.40**) + D (r, 0.38**) − PA (r, − 0.25**)					+ EE*** + D*** − PA**					1993
Deary et al. [48]	+ EE (r, 0.56**) + D (r, 0.40**) − PA (r, − 0.35**)	+ PA (r, 0.27**)	− D (r, − 0.21**)	+ PA (r, 0.20**)	+ PA (r, 0.15**)						1996
Deary et al. [112]	+ EE (r, 0.76***) + D (r, 0.71***)										1996
Mills et al. [60]	+ EE (T1) (r, 0.50**) + EE (T2) (r, 0.43**) + D (T1) (r, 0.34**) + D (T2) (r, 0.23**) − rPA (T1) (r, − 0.31**) − rPA (T2) (r, − 0.29**)	− EE (T1) (r, − 0.24**) − EE (T2) (r, − 0.22**) + rPA (T1) (r, 0.48**) + rPA (T2) (r, 0.42**)	− EE (T1) (r, − 0.24**) − EE (T2) (r, − 0.23*) − D (T1) (r, − 0.36**) − D (T2) (r, − 0.25*)	− EE (T1) (r, − 0.37*) − EE (T2) (r, − 0.12*) + rPA (r, 0.19*)			− EE (T1) (β, − 0.26*) + rPA (T1) (β, 0.45*)	− D (T1) (β, − 0.31*)			1998
Zellars et al. [113]	+ EE (r, 0.50*) + D (r, 0.23*) − rPA (r, 0.23*)	− EE (r, − 0.25*) −D (r, − 0.30*) − rPA (r, − 0.28*)	− EE (r, − 0.29*) −D (r, − 0.37*) − rPA (r, − 0.19*)		−rPA (r, − 0.24*)	+ EE (β, 0.32***)	− D (β, − 0.20***) − rPA (β, − 0.18**)	− D (β, − 0.32***)		−D (β, −0.11*) −rPA (β, −0.25***)	2000
Zellars et al. [53]	+ EE (r, 0.48**) + D (r, 0.38**) + rPA (r, 0.30**)	−EE (r, −0.24**) −D (r, −0.19**) −rPA (r, −0.37**)	−EE (r, −0.24**) −D (r, −0.39**) −rPA (r, −0.29**)			+ EE (β, 0.34**) + D (β, 0.24**)	−rPA (β, −0.21**)	− D (β, −0.28**) −rPA (β, −0.11**)			2001
De Vries et al. [114]		−EE (r, −0.34**)		−EE (r, −0.13**)							2002
McManus et al. [50]	+ EE (r, 0.47**) + D (r, 0.38**) + rPA (r, 0.30**)	− EE (r, − 0.24**) − D (r, − 0.19**) − rPA (r, − 0.37**)				+ EE (β, 0.37**) + D (β, 0.30**)	− rPA (β, − 0.28**)				2004
Zellars et al. [52]	T1 + EE (T2) (r, 0.233***) T1 + D (T2) (r, 0.103***) T2 + EE (T2) (r, 0.378***) T2 + D (T2) (r, 0.235***) T2 + PA (T2) (r, 0.090***)	T1−EE (T2) (r, − 0.190***) T1−D (T2) (r, − 0.137***) T1−PA (T2) (r, − 0.077***) T2−EE (T2) (r, − 0.262***) T2−D (T2) (r, − 0.171***) T2−PA (T2) (r, − 0.152***)	T1−D (T2) (r, − 0.240***) T1−PA (r, − 0.091***) T2−EE (T2) (r, − 0.094***) T2−D (T2) (r, − 0.322***) T2−PA (r, − 0.082***)	T1−EE (T2) (r, − 0.088**) T1−D (T2) (r, − 0.121***) T2−EE (r, − 0.129***) T2−D (r, − 0.165***)	T1 + PA (r, 0.096**) T2 + PA (r, 0.127***)						2004
Cano-García et al. [115]						+ EE (β, 0.71***)		−D (β, − 0.37*) + PA (β, 0.58***)			2005
Burke et al. [116]		−EE (r, − 0.19*) −C (r, − 0.19*)	−EE (r, − 0.12*) −C (r, − 0.18*)	−EE (r, − 0.23*) −C (r, − 0.31*)			− EE (β, − 0.05*)		− EE (β, − 0.18***) − C (β, − 0.28***)		2006
Goddard et al. [57]	+ B (r, 0.45***)	− B (r, − 0.16***)	− B (r, − 0.24***)	− B (r, − 0.12*)		+ B (β, 0.24***)		− B (β, − 0.08***)	− B (β, − 0.09***)	+ B (β, 0.11***)	2006
Langelaan et al. [117]	+ EE (r, 0.50**) + C (r, 0.48**)	+ EE (r, − 0.33**) + C (r, − 0.37**)				+ EE (β, 0.81***)					2006
Mostert et al. [118]						+ EE (T4) (β, 0.19**) − PA (β, − 0.17*)					2006
Bahner et al. [119]	+ EE (r, 0.44**) + C (r, 0.39**)	− EE (r, − 0.24**) − C (r, − 0.20**) + PA (r, 0.23**)	− EE (r, − 0.24**) − C (r, − 0.33**) + PA (r, 0.41**)	+ PA (r, 0.34**)	+ PA (r, 0.23**)	+ EE (β, 0.32**) + C (β, 0.30**)	− EE (β, − 0.13*)	− C (β, − 0.24**) + PA (β, 0.28**)	+ PA (β, 0.24**)		2007
Ghorpade et al. [120]		− EE (r, − 0.213***) + PA (r, 0.221***)	− EE (r, − 0.135***) − D (r, − 0.438***) + PA (r, 0.356***)	− D (r, − 0.164***) + PA (r, 0.307***)	+ PA (r, 0.251***)		− EE (M2; β, − 1.600**; M7; β, − 1.563**) + PA (M2; β, − 1.479***; M7; β, − 0.961**)	− EE (M4; β, − 2.685**) − D (M4; β, − 3.134***; M7; β, − 2.732***) − PA (M4; β, − 3.209***; M7; β, − 1.903***)	+ PA (M3; β, − 2.119***; M7; β, − 1.398***)	+ EE (M7; β, 1.960**) + PA (M5; β, − 2.011***)	2007
Kim et al. [121]								− D (β, − 3.77*)			2007
Teven, [122]	+ EE (r, 0.50*) + D (r, 0.39*) + rPA (r, 0.38*)	− D (r, − 0.13*) − rPA (r, − 0.40*)	− D (r, − 0.37*)	− EE (r, − 0.41*) − D (r, − 0.54*)	+ EE (r, 0.08*)						2007
Leon et al. [123]	+ EE (r, 0.38**) + D (r, 0.38**) − PA (r, − 0.31**)	− EE (r, − 0.18**) − D (r, − 0.14*) + PA (r, 0.30**)									2008
Chung et al. [124]	− rPA (r, − 0.442***)					− PA (β, − 0.395**)					2009
De Hoogh et al. [125]	+ EE (r, 0.40**) + C (r, 0.38**) − PA (r, − 0.17*)	+ C (r, 0.21**)	− C (r, − 0.28**)	− C (r, − 0.24**) + PA (r, 0.41**)		+ B (β, 0.35**) + EE (β, 0.38**) + C (β, 0.24*)					2009
Gandoy− Crego et al. [126]	+ EE (r, 0.41***) + D (r, 0.50***)					+ EE (β, 0.20**) + D (β, 0.34***)					2009
Kim et al. [127]						+ EE (β, 0.33***) − PA (β, − 0.27**)	− EE (β, − 0.20*) + PA (β, 0.26*)		+ D (β, 0.24*)		2009
Taormina et al. [128]	+ B (S1) (r, 0.56**) + B (S2) (r, 0.65**)										2009
Barford et al. [129]				− EE (r, − 0.25**)					− EE (S1) (β, − 0.20**)		2010
Perry et al. [51]						+ EE (β, 0.27**) + D (β, 0.21*) − PA (β, − 0.27*)	+ PA (β, 0.27*)	− D (β, − 0.23*)	+ PA (β, 0.25*)		2010
Ghorpade et al. [130]	+ EE (r, 0.35*) + D (r, 0.24*) − PA (r, − 0.23*)	− EE (r, − 0.20*) + PA (r, 0.30*)		− D (r, − 0.30*) + PA (r, 0.32*)	− D (r, − 0.19*) − PA (r, − 0.20*)	+ EE (β, 0.20*) + D (β, 0.23*) + PA (β, 0.20*)	+ EE (β, 0.25*) + PA (β, 0.18*)		+ EE (β, 0.23*) + D (β, 0.25*) + PA (β, 0.22*)	+ EE (β, 0.18*) + D (β, 0.18*)	2011
Hudek− Knežević et al. [59]	+ EE (T1) (r, 0.39*) + EE (T2) (r, 0.34*)					+ EE (T2) (M1; β, 0.31***; M2; β, 0.15**)					2011
Salami, [131]		− EE (r, − 0.21**) + PA (r, 0.22**)	− EE (r, − 0.14*) − D (r, − 0.44**) + PA (r, 0.36**)	− D (r, − 0.16**) + PA (r, 0.31**)	+ PA (r, 0.25**)		− EE (β, − 1.24*)	+ PA (β, 2.02**)			2011
Sterud et al. [61]			− rPA (T2) (r, − 0.27**)		− rPA (T2) (r, − 0.19*)			− rPA (T2) (β, − 0.28**)		− rPA (T2) (β, 0.10*)	2011
Armon et al. [54]	+ B (r, 0.35*)										2012
Zimmerman et al. [132]	+ B (T1) (r, 0.25*) + B (T2) (r, 0.20*) + EE (T1) (r, 0.28*) + EE (T2) (r, 0.21*)	+ B (T1) (r, 0.28*) + B (T2) (r, 0.25*) + EE (T1) (r, 0.23*) + EE (T2) (r, 0.22*)	− B (T1) (r, − 0.07*) − EE (T1) (r, − 0.21*) − EE (T2) (r, − 0.18*)	− B (T1) (r, − 0.12*) − B (T2) (r, − 0.12*) − EE (T2) (r, − 0.09*)	+ EE (T1) (r, 0.08*) + EE (T2) (r, 0.07*)	+ B (T1) (β, 0.21*) − EE (T1) (β, − 0.18*) − EE (T2) (β, − 0.21*)	− B (T1) (β, − 0.16*)	+ B (T1) (β, 0.16*) − EE (T1) (β, − 0.83*) − EE (T2) (β, − 0.48*)	− B (T1) (β, − 0.21*) + EE (T1) (β, 0.34*) + EE (T2) (β, 0.18*)		2012
De la Fuente Solana et al. [133]	+ EE (r, 0.50***) + D (r, 0.39***) − PA (r, − 0.45***)	− EE (r, − 0.36***) − D (r, − 0.29***) + PA (r, 0.43***)	− EE (r, − 0.45***) − D (r, − 0.45***) + PA (r, 0.33***)	− EE (r, − 0.34***) − D (r, − 0.31***) + PA (r, 0.51***)	+ PA (r, 0.14***)	+ EE (β, 0.50***) + D (β, 0.18***)	+ PA (β, 0.30***)	− EE (β, − 0.48***) − D (β, − 0.27***) + PA (β, 0.22**)	+ PA (β, 0.40***)		2013
Garbarino et al. [134]							+ PA (M2; β, 0.15**; M3; β, 0.14**; M4; β, 0.15**; M5; β, 0.14**)	− D (M2; β, − 0.23***; M3; β, − 0.24***; M4; β, − 0.24***; M5; β, − 0.24***) + PA (M2; β, 0.26***; M3; β, 0.27***; M4; β, 0.26***; M5; β, 0.27***)	+ PA (M2; β, 0.23***; M3; β, 0.24***; M4; β, 0.23***; M5; β, 0.24***)	+ PA (M2; β, 0.17**; M3; β, 0.16**; M4; β, 0.17**; M5; β, 0.16**)	2013
Hurt et al. [135]	+ EE (r, 0.380***) + C (r, 0.254**) − PA (r, − 0.338***)	− C (r, − 0.315***) + PA (r, 0.413***)	+ PA (r, 0.322***)	− C (r, − 0.208*) + PA (r, 0.369***)							2013
Lin et al. [136]	+ EE (r, 0.432***) + D (r, 0.266***) − PA (r, − 0.197**)	− EE (r, − 0.174**) + PA (r, 0.137*)				+ EE (β, 0.25***)					2013
Gan et al. [56]	+ B (r, 0.418**)	− B (r, − 0.173*)		− B (r, − 0.181*)	− B (r, − 0.237**)						2014
Reinke et al. [137]	+ EE (T1) (r, 0.18)^†^ + EE (T2) (r, 0.20)^†^ + EE (T3) (r, 0.20)^†^ + C (T1) (r, 0.21)^†^ + rPA(T1) (r, 0.23)^†^ + rPA(T2) (r, 0.18)^†^ + rPA(T3) (r, 0.17)^†^	− EE (T1) (r, − 0.25)^†^ − EE (T2) (r, − 0.21)^†^ − EE (T3) (r, − 0.22)^†^ − C (T1) (r, − 0.36)^†^ − C (T2) (r, − 0.25)^†^ − C (T3) (r, − 0.24)^†^ − rPA(T1) (r, − 0.35)^†^ − rPA(T2) (r, − 0.20)^†^ − rPA(T3) (r, − 0.17)^†^		− rPA(T1) (r, − 0.40)^†^ − rPA(T2) (r, − 0.33)^†^ − rPA(T3) (r, − 0.30)^†^			− EE (T3)^†^		− PA (T1)^†^ − PA (T2)^†^ − PA (T3)^†^		2014
Taycan et al. [138]	+ EE (r, 0.43***) + D (r, 0.35***) − PA (r, − 0.28***)	− EE (r, − 0.20*) − D (r, − 0.25**) + PA (r, 0.27***)				+ EE (β, 0.36***) + D (β, 0.30***)					2014
Yilmaz, [139]		− EE (r, − 0.14*) − D (r, − 0.17**) − rPA (r, − 0.28**)	− EE (r, − 0.17**) − D (r, − 0.25**) − rPA (r, − 0.38**)	− EE (r, − 0.18**) − D (r, − 0.33**) − rPA (r, − 0.37**)	− D (r, − 0.15**) − rPA (r, − 0.45**)						2014
Cañadas− De la Fuente et al. [140]	+ EE (r, 0.58***) + D (r, 0.41***) − PA (r, − 0.41***)	− EE (r, − 0.41***) − D (r, − 0.30***) + PA (r, 0.45***)	− EE (r, − 0.37***) − D (r, − 0.48***) + PA (r, 0.42***)	− EE (r, − 0.30***) − D (r, − 0.37***) + PA (r, 0.53***)	− EE (r, − 0.11**) − D (r, − 0.19***) + PA (r, 0.24***)	+ EE (β, 0.63***) + D (β, 0.13***) − PA (β, − 0.12**)	− EE (β, − 0.24***)	− EE (β, − 0.24***) − D (β, − 0.31***) + PA (β, 0.22***)	− D (β, − 0.11**) + PA (β, 0.38***)		2015
Srivastava et al. [141]						+ B (St1; β, 0.378**; St2; β, 0.390**)					2015
Ang et al. [142]	+ B (r, 0.10**)										2016
Iorga et al. [143]						+ EE^†^ + D^†^ + rPA^†^	− EE^†^ − D^†^ − rPA^†^	+ EE^†^ + D^†^ + rPA^†^	+ EE^†^ + D^†^ + rPA^†^	+ rPA^†^	2016
Vaulerin et al. [144]	+ EE (r, 0.19**)										2016
Zhou et al. [145]	+ EE (r, 0.506*) + D (r, 0.368*)	− D (r, − 0.401*) − PA (r, − 0.372*)	− D (r, − 0.588**) + PA (r, 0.390*)	− EE (r, − 0.387*) − D (r, − 0.348*) − PA (r, − 0.554***)			− D (β, − 0.380*)		+ PA (β, 0.389*)		2016
De la Fuente− Solana et al. [146]	+ EE (r, 0.472***) − D (r, 0.298**) + PA (r, − 0.270**)	− EE (r, − 0.293***) − D (r, − 0.229*) − PA (r, − 0.361***)	− EE (r, − 0.380***) − D (r, − 0.583***) + PA (r, 0.531***)	− EE (r, − 0.343***) − D (r, − 0.405***) + PA (r, 0.612***)	− EE (r, − 0.276**) − D (r, − 0.278**) + PA (r, 0.286**)						2017
Geuens et al. [147]	+ EE (r, 0.56***) − PA (r, − 0.28***)	− EE (r, − 0.25***) + PA (r, 0.47***)	− D (r, − 0.25***) + PA (r, 0.37***)	+ PA (r, 0.22***)		+ EE (T1) (β, − 0.56***) + EE (T2) (β, − 0.54***) − PA (T2) (β, − 0.23**)		− D (T1) (β, − 0.22*)			2017
Iorga et al. [148]	+ EE (r, 0.475***) + D (r, 0.231*) − PA (r, − 0.456***)	− EE (r, − 0.330***) − D (r, − 0.221*) + PA (r, 0.388***)	− D (r, − 0.313***) + PA (r, 0.368***)	− D (r, − 0.239**) + PA (r, 0.315***)	− D (r, − 0.218*) + PA (r, 0.363***)	+ EE (β, 0.377***)					2017
Lovell et al. [149]						+ EE (β, 0.618***) + D (β, 0.220***) − PA (β, − 0.124*)			− D (β, − 0.171***) + PA (β, 0.172***)	+ PA (β, 0.094*)	2017
Ntantana et al. [150]						+ EE***	− EE***				2017
Al Shbail et al. [151]	− B (r, 0.382**) − EE (r, 0.335**) + rPA (r, 0.346**)	− B (r, − 0.203*) − EE (r, − 0.171*)	− B (r, − 0.219**) − EE (r, − 0.223**) − rPA (r, − 0.316**)			+ B (β, 0.325**) + EE (β, 0.299**)		+ rPA (β, − 0.236**)			2018
Bergmüller et al. [152]	+ B (T1; r, 0.24**) + B (T2; r, 0.29**)										2018
Bianchi et al. [153]	+ B (r, 0.589***)					+ B (β, 0.219**)					2018
Bianchi, [19]						− EE (β, − 0.374**) − D (β, − 0.282*)		− EE (β, − 0.261*) − D (β, − 0.440***)			2018
Harizanova et al. [49]	+ B (r, 0.63***)	− B (r, − 0.33***)				+ B (β, 0.40***)	− B (β, − 0.11*)				2018
Hildenbrand et al. [58]	+ B (r, 0.56*)	− B (r, − 0.24*)				+ B (β, 0.43***)	− B (β, − 0.06***)				2018
Iorga et al. [154]	+ EE (r, 0.266*) − PA (r, − 0.289*)	+ PA (r, 0.458**)	− D (r, − 0.272*) + PA (r, 0.374***)	− D (r, − 0.318**) + PA (r, 0.257*)	+ PA (r, 0.341**)		+ PA (β, 0.480)^†^				2018
Tang et al. [155]	+ EE (r, 0.76**) + D (r, 0.59**) − PA (r, − 0.49**)	− EE (r, − 0.45**) − D (r, − 0.41**) + PA (r, 0.55**)				+ EE (β, 0.575**) + D (β, 0.514**)	− EE (β, − 0.096*) − D (β, − 0.134**)				2018
Tatalović Vorkapić et al. [156]			− D (r, − 0.24***)	− D (r, − 0.19**)							2018
Yao et al. [157]	+ B (r, 0.35**)	− B (r, − 0.23**)				+ B (β, 0.587***)					2018
Zaninotto et al. [158]	+ EE (r, 0.35**) + D (r, 0.30**)										2018
Bahadori et al. [159]	− B (r, − 0.275**)	− B (r, − 0.034**)	− B (r, − 0.260**)	− B (r, − 0.283**)	− B (r, − 0.187**)	− B (M1; β, − 0.388**; M2; β, − 0.142*)		− B (M1; β, − 0.359**; M2; β, − 0.523*)		− B (M1; β, − 0.310**; M2; β, − 0.092*)	2019
Brown et al. [160]	− EEf (r, − 0.380**) − EEs (r, − 0.321**) − Df (r, − 0.126*) − rPAf (r, − 0.370**) − rPAs (r, − 0.428**)	− EEf (r, − 0.162**) + rPAf (r, 0.246**) + rPAs (r, 0.490**)	+ EEf (r, 0.162**) + EEs (r, 0.129*) − Ds (r, − 0.265**) + rPAf (r, 0.119*)	+ rPAf (r, 0.162**) + rPAs (r, 0.394**)	+ Df (r, 0.185**) + rPAf (r, 0.162**) + rPAs (r, 0.326**)	− B (β, − 0.66***)	− B (β, − 0.31***)		− B (β, − 0.284***)		2019
Castillo− Gualda et al. [62]	+ B (r, 0.36**)										2019
De la Fuente− Solana et al. [161]						+ EE (β, 0.092**)		− D (β, − 0.187***) − PA (β, − 0.164**)			2019
De Looff et al. [55]	+ B (r, 0.20***)	− B (r, − 0.14***)	− B (r, − 0.15***)	− B (r, − 0.20***)	− B (r, − 0.18***)						2019
Farfán et al. [162]	+ B (r, 0.42**)	− B (r, − 0.26**)				+ B (M4; β, 0.16**; M5; β, 0.16*)					2019
Khedhaouria et al. [163]	+ EE (r, 0.353**) + D (r, 0.303**) − PA (r, − 0.376**)										2019
Pérez− Fuentes et al. [17]	+ EE (r, 0.59***) + D (r, 0.42**) − PA (r, − 0.39**)		− D (r, − 0.40**) + PA (r, 0.35*)	− D (r, − 0.32*) + PA (r, 0.36*)							2019
Ye et al. [164]	Study 1: + EE (r, 0.43***) + D (r, 0.23***) − rPA (r, − 0.25***) Study 2: + EE (T1) (r, 0.34**) − EE (T2) (r, 0.32*) + D (T1) (r, 0.28*) − PA (T1) (r, − 0.40**) − PA (T2) (r, − 0.29*)	Study 1: − EE (r, − 0.23***) − D (r, − 0.16*) + rPA (r, 0.32***) Study 2: + PA (T1) (r, 0.29*)	Study 1: − EE (r, − 0.31***) − D (r, − 0.43***) (r, − 0.40**) + rPA (r, 0.51***) Study 2: − EE (T2) (r, − 0.38**) − D (T1) (r, − 0.39**) + PA (T1) (r, 0.36***) + PA (T2) (r, 0.27*)	Study 1: − EE (r, − 0.20**) − D (r, − 0.35***) + rPA (r, 0.38***)	Study 1: − D (r, − 0.23***) + rPA (r, 0.44***)	Study 1: + EE (β, 0.62***) + D (β, 0.18*) − PA (β, − 0.16*)		Study 1: − EE (β, − 0.32*) − D (β, − 0.39**) + PA (β, 0.46***) Study 2: − EE (β, − 0.49*) − D (β, − 0.48*)	Study 1: − D (β, − 0.22*) + PA (β, 0.22*)	Study 1: + PA (β, 0.43***)	2019
Banasiewicz et al. [165]		− EE (r, − 0.19**)	− EE (r, − 0.18**)	− EE (r, − 0.17**)				− EE (M1; β, − 0.15*; M2; β, − 0.16**; M3; β, − 0.14*; M4; β, − 0.14*)			2020
Bhowmick et al. [166]			− B (r, − 0.250**)	− B (r, − 0.167**)	− B (r, − 0.231**)			− B (β, − 0.33*)		− B (β, − 0.45*)	2020
De Vine et al. [167]	+ EE (r, 0.418*) + C (r, 0.287*) − PA (r, − 0.208*)	− EE (r, − 0.278*) + C (r, 0.291*) + PA (r, 0.274*)		− EE (r, − 0.303*) − C (r, − 0.250*) + PA (r, 0.275*)	− EE (r, − 0.265*) − C (r, − 0.170*) + PA (r, 0.274*)	+ EE (β, 0.365***) + C (β, 0.196*)		+ EE (β, 0.220**) + C (β, 0.222*)	+ PA (β, 0.221**)	+ EE (β, − 0.193*)	2020
Dionigi, [168]	+ B (r, 0.285***)	− B (r, − 0.180***)	− B (r, − 0.353***)	− B (r, − 0.355***)		+ B (β, 0.215***)			− B (β, − 0.300***)		2020
Farfán et al. [169]	− EE (r, − 0.209**)	− EE (r, − 0.239**)				+ EE***	− EE***				2020
Liu et al. [170]	+ EE (r, 0.28**) − PA (r, − 0.17*)	+ PA (r, 0.24**)	+ PA (r, 0.26**)	− EE (r, − 0.20*) + PA (r, 0.38**)	+ PA (r, 0.21**)	+ EE (β, 0.24*)			+ PA (β, 0.31***)		2020
Mahoney et al. [171]	+ EE (r, 0.16*)		− EE (r, − 0.23**) − D (r, − 0.34**) + PA (r, 0.27**)	− EE (r, − 0.37**) − D (r, − 0.43**) + PA (r, − 0.23**)					− EE (β, 0.362**) − D (β, 0.362**)		2020
Malka et al. [172]	+ EE (S1) (r, 0.343***) + EE (S2) (r, 0.331***)										2020
Tasic et al. [173]	+ B (r, 0.452***)			− B (r, − 0.202*)							2020
Bianchi et al. [174]	+ B (S1; r, 0.552***) (S2; r, 0.562***) (S3; r, 0.642***)					+ B (S1; β, 0.373***; S2, β, 0.318***; S3, β, 0.409***)					2021
Fuente-Solana et al. [175]	+ EE (r, 0.610**) + D (r, 0.372**) − PA (r, − 0.285**)	− EE (r, − 0.407**) − D (r, − 0.351**) + PA (r, 0.422**)	− EE (r, − 0.276**) − D (r, − 0.439**) + PA (r, 0.269***)	− EE (r, − 0.318**) − D (r, − 0.441**) + PA (r, 0.497**)	+ PA (r, 0.280**)	+ EE (β, 0.350***)	+ PA (β, 0.268**)	− D (β, − 0.305**)	− D (β, − 0.236*) + PA (β, 0.391***)		2021
Fuente–Solanaet al. [176]	+ EE (r, 0.565**) + D (r, 0.432**) − PA (r, − 0.270**)	− EE (r, − 0.304**) − D (r, − 0.199***) + PA (r, 0.419**)	− EE (r, − 0.323**) − D (r, − 0.377**) + PA (r, 0.332**)	− EE (r, − 0.290**) − D (r, − 0.309**) + PA (r, 0.371**)	+ PA (r, 0.407**)	+ EE (β, 0.250**) + D (β, 0.324***)		− D (β, − 0.242**) + PA (β, 0.205**)		+ PA (β, 0.324***)	2021

***p < 0.001; **p < 0.01; *p < 0.05^†^, significant value, but the degree of significance is not reported in the original paper; -, negative; + , positive; B, Burnout; C, Cynicism; D, Depersonalization; EE, Emotional Exhaustion; f, frequency; PA, Personal/Professional Accomplishment; rPA, Reduced Personal or Professional Accomplishment; s, severity; S1, Sample 1; S2, Sample 2; S3, Sample 3; St, Step; T1, Time 1; T2, Time 2; T3, Time 3; T4, Time 4. When the index of r or β is not reported, it means that it was not present in the original paper

r < 0.19, very weak; 0.20–0.39, weak; 0.40–0.59, moderate; 0.60–0.79 strong; > 0.80, very strong correlation [205]

### Extraversion

A negative association between Extraversion and job burnout was reported (range, r from − 0.034** to − 0.33***; β from − 0.06*** to − 0.31***). Longitudinal studies also suggest the role of Extraversion as a protective factor against burnout and its dimension of Exhaustion (B; β, − 0.16*; EE; β, − 0.26*). As seen previously, the Extraversion trait has been identified as the intensity of social interaction and the level of self-esteem of individuals [32]. People with higher levels of extraversion appear positive, cheerful, optimistic, and have more likely to experience positive emotions [206]. This positive view of their level of job-related self-efficacy [207], often associated with the interpersonal bonds they tend to create [208] can protect outgoing individuals from experiencing high levels of emotional exhaustion. On the contrary, introverted individuals tend to experience greater feelings of helplessness and lower levels of ambition [204], which instead results in a risk factor for job burnout. Although the negative association is the most frequent, some studies have found a directly proportional association between Burnout and Extraversion [54], Cynicism [127, 173], and reduced Professional Accomplishment [50, 60, 62, 143, 146, 159]. Again, the authors do not provide reasons for this discordant result from the literature.

### Neuroticism

A positive association between Neuroticism and job burnout was reported (range, r from 0.10** to 0.642***; β from 0.16** to 0.587***). Longitudinal studies also suggest a role of Neuroticism as a predictor of Burnout and its extent of Exhaustion, while predicting a decrease in Professional Accomplishment (B; β, 0.21*; EE; β, 0.31***; β, 0.15**; β, 0.19**; PA; β, − 0.23**). As seen previously, it is possible to define Neuroticism as the inability of people to control their impulses and manage their emotional balance. Neurotic people experience a series of feelings of insecurity, anxiety, anger, and depression [25, 76, 204] that they try to manage through maladaptive coping strategies, such as delay or denial [29, 34]. These characteristics of the personality trait of Neuroticism would interfere with job functioning and satisfaction, operating a negative "filter" that magnifies the impact of adverse events (see [209]) and constitutes a significant risk factor for job burnout [8, 174]. Feelings of anxiety and nervousness could lead them more easily to experience higher levels of emotional exhaustion, and by focusing on more aspects of their work, they are more likely to manifest depersonalization. Although most studies report a positive association between Neuroticism and Burnout [164], Burnout [159, 169], Depersonalization [133, 159], and reduced Professional Accomplishment [60, 62, 126]. Ye and colleagues [164] tie this result to the Chinese cultural situation, whereby the observed greater sense of responsibility and discipline could reduce the effects of extroversion on job burnout. Farfán and colleagues [169], on the contrary, link this result to the tendency of the neurotic personality trait to use rationalization as a defense against job burnout. Unlike most of the studies included in this review, some results show a negative association between Neuroticism and Burnout [159, 164], Emotional exhaustion, and Depersonalization [155]. Furthermore, a study indicates that Neuroticism is positively associated with reduced Personal/Professional Accomplishment [131]. Finally, in the longitudinal study by Armon and colleagues [54], Neuroticism even seems to protect against Emotional exhaustion. The authors explain the association over time of Neuroticism with job burnout as due to an underrepresentation in the measurement scales used or the moderating effect of gender on these associations [159].

### Openness

A negative association between Openness and job burnout was reported (range, r from − 0.18*** to − 0.237**; β from − 0.092* to − 0.45*). Longitudinal studies have suggested the role of Openness as a protective factor of reduced Professional Accomplishment (rPA; β, 0.10*). As seen previously, individuals with high levels of Openness tend to be more intellectually curious about novelty and open-minded and have a predisposition to independence [35, 76, 202]. These characteristics protect individuals from experiencing discomfort, experiencing novelty and failures as opportunities [203], and protecting them from job burnout from emotional exhaustion. Conversely, when faced with stressors at work, less open individuals can adopt quick but suboptimal strategies, such as depersonalization [8]. Although most of the studies found a negative relationship between Openness and job burnout, five studies found a positive correlation between Openness and Emotional exhaustion [54, 122] and Depersonalization [159], while negative with Personal/Professional Accomplishment [62, 131, 159]. The authors do not provide reasons for this discordant result from the literature. Other studies instead have found a positive association between Openness and all dimensions of Burnout [116]: Exhaustion [131, 173], Depersonalization [131], and reduced Personal/Professional Accomplishment [142]. Finally, the longitudinal study by Ghorpade and colleagues [120] attributes Openness to the role of the positive predictor of Emotional exhaustion. According to the authors, this result could be attributed to the work of the professors (Professors) which, requiring a greater openness to listening to students' different problems and encouraging different positions in them, could increase emotional exhaustion.

The findings of most of the studies reviewed indicate that individuals who have higher levels of neuroticism and lower agreeableness, conscientiousness, extraversion, and openness to experience are more prone to experiencing job burnout. However, the few studies that show other results than this theoretical line cannot explain the conflicting results. Some authors adduce these results to a measurement bias (e.g., [159]) or sample characteristics (e.g., [120]) but fail to explain the reason for this relationship and believe that it is due to further variables to be explored.

### Limitations

Although the literature review was conducted as rigorously as possible, the search strategy was limited to four scientific search engines. Furthermore, it was impossible to find all the relevant studies if the search terms were not mentioned in the articles' titles, abstracts, or keywords. Therefore, some related papers might be missed due to the selected terms. Furthermore, the search included only studies published in English, thus excluding relevant studies in other languages. Additionally, gray literature was not included in the study, and therefore, it may not have been considered essential data contained in non-peer-reviewed studies, unpublished theses, and dissertation studies. Furthermore, one of the exclusion criteria was the journal ranking of SCImago. Although this is a widely accepted and recognized measure to reduce the possibility of including in systematic reviews papers that do not meet certain quality indices [47], they may not have been considered relevant data. In addition, the Big Five model [46] was used as a conceptual model of reference to compare the results of the studies on job burnout. Studies that did not include the Big Five models or that explored the relationship between Burnout and personality disorders (e.g., Antisocial Personality Disorder, Narcissistic Personality Disorder, Borderline Personality Disorder, etc.) were therefore not examined in this study. Restricting studies to a single conceptual model of personality was necessary to focus the review, but at the same time, it limited our investigation. Furthermore, the heterogeneity of the study samples' work type, burnout measurement tools, and personality traits prevented comparing results across studies. Finally, despite precautions to reduce selection bias, confounding, and measurement bias, no studies have addressed reverse causality problems in the relationship between personality traits and burnout. Although the cross-sectional research design does not allow us to investigate the causal links between personality and burnout, an answer to the existence of this link is offered by the longitudinal studies included in the review. This type of study demonstrates that personality traits play a role in the development of burnout, but future research must investigate this relationship, especially with the help of longitudinal studies that can reduce the problems related to reverse causality.

## Conclusions

The findings obtained in the present review highlight the importance of examining the role of personality traits in the development of job burnout syndrome. At the same time, it is possible to observe how scientific evidence places us in front of a picture that is not fully defined. In line with Guthier's meta-analysis [200], the findings of this review highlight the need for expanding job stress theories focusing more on the role that personality plays in burnout.

I am convinced of the value of this review in directing future empirical research on job burnout, especially in the light of new approaches to burnout as a multi-component factor (see [210, 211]). Even more future research will have the task of encouraging the use of methodologies that evaluate personality traits in work contexts. An assessment of personality traits and continuous monitoring of occupational stress levels (e.g., [212]) could help identify the people who are most likely to develop burnout syndrome to prevent or limit its damage. Future research should improve understanding and intervention on burnout, too often limited by universal approaches that have neglected the uniqueness of the antecedents of burnout [213]. Some traits related to burnout predict work outcomes such as job performance, job satisfaction, and turnover [203, 214–218]. It is, therefore, necessary to investigate the antecedents of Burnout to provide implications practices for jobs and organizations.

## Data Availability

As this is a systematic review of the literature, this study indicates the information to obtain all data analyzed in the databases used. However, the datasets used during the current study remain available from the corresponding author upon reasonable request.
